# Synergy of cancer immunotherapy and radiotherapy

**DOI:** 10.18632/aging.100730

**Published:** 2015-03-24

**Authors:** Aaron S. Mansfield, Sean S. Park, Haidong Dong

**Affiliations:** ^1^ Division of Medical Oncology, Department of Oncology, Mayo Clinic, Rochester, MN 55905, USA; ^2^ Department of Radiation Oncology, Department of Oncology, Mayo Clinic, Rochester, MN 55905, USA; ^3^ Department of Immunology, Department of Oncology, Mayo Clinic, Rochester, MN 55905, USA; ^4^ Department of Urology, Department of Oncology, Mayo Clinic, Rochester, MN 55905, USA

Radiotherapy is a cornerstone of cancer care in non-metastatic patients. It has been incorporated into the treatment regimens of major tumor histologies with curative intent, and is broadly applied for almost any tumor type in the metastastic setting to alleviate symptoms. The cytotoxic effects of radiotherapy have primarily been attributed to double strand DNA damage; however, recent evidence suggests that its immunomodulatory effects can contribute to its therapeutic efficacy. This synergistic potential from combining radiotherapy and immunotherapy may have a role in patients with locally advanced or metastatic cancer.

Immune checkpoint inhibitors have been developed to blunt tumor-based immunosuppressive signals. Cytotoxic T-lymphocyte-associated protein 4 (CTLA-4) and programmed cell death protein 1 (PD-1) are protein receptors expressed on T-cells whose engagement with their ligands (B7-1 or B7-2, and B7-H1 or B7-H2 respectively) limits T cell activity. The CTLA-4 inhibitor (ipilimumab) and the PD-1 inhibitors (pembrolizumab and nivolumab) have been approved by the FDA for the treatment of metastatic melanoma (all of the agents) and squamous cell lung cancer (nivolumab). Interestingly, an abscopal or off-target effect has been reported in a patient with metastatic melanoma where the addition of radiotherapy to ipilimumab led to tumor response in non-radiated lesions in addition to the radiated lesion [1]. This case report has stimulated significant excitement for the synergism between radiotherapy and immunotherapy in the clinical setting.

A series of preclinical experiments have further developed the rationale for combining radiotherapy and immunotherapy. Radiotherapy has the potential to overcome many of the mechanisms of tumor immune escape through the release of immunogenic private antigens, enhanced tumor expression of MHC-I, release of the TLR4 agonist HMGB-1, and the generation of tumor specific cytotoxic T cells (as reviewed in Kalbasi et al. [2]). Radiotherapy also increases the expression of the immunosuppressive protein B7-H1 (aka PD-L1) on tumor cells [3]. Accordingly, when radiotherapy has been combined with inhibitors of PD-1 in malignant melanoma or breast cancer xenografts [4], an orthotopic glioblastoma xenograft model [5], or an inhibitor of PD-L1in colon cancer or breast cancer xenografts [3], control of the growth of the radiated tumor and survival of the mice were improved when compared to radiotherapy or the immune checkpoint inhibitors alone. In one study, this synergistic effect was dependent upon CD8+ T cells [3]; however, another study identified both tumor-specific cytotoxic T cells and antibodies [4]. In a separate study where mice received a CTLA-4 inhibitor and radiotherapy, PD-L1 was one of the most upregulated genes in resistant tumors, and addition of a PD-L1 inhibitor significantly improved responses of resistant tumors [6]. In our own experiments, we investigated the influence of PD-1 expression on the systemic antitumor response induced by radiotherapy in melanoma and renal cell carcinoma models. We compared the radiotherapy-induced antitumor response in PD-1-expressing wild-type and PD-1-deficient knockout mice. We found that survival was better in the PD-1 knockout mice than the wild-type mice. The combination of radiotherapy and a PD-1 inhibitor in wild-type mice resulted in a synergistic, near complete regression of the irradiated primary tumors as compared to either treatment alone. This combination also elicited significant responses in non-irradiated, secondary tumors outside the radiotherapy field, consistent with an abscopal effect. The combinatory effect of radiotherapy and PD-1 blockade was 1) tumor-antigen specific, 2) not dependent on specific tumor histology, and 3) not limited to certain host genetic background [7]. Additionally, we found that combined blockade of PD-1 and CTLA-4 with radiotherapy demonstrated significant therapeutic effect in both irradiated and non-irradiated tumor of large burden [7]. We believe that the clinical case reports of abscopal effects, and the preclinical models summarized above provide a strong rationale for testing the combination of radiotherapy with immune checkpoint inhibitors, specifically PD-1/PD-L1 inhibition, in clinical trials for humans.

There are significant challenges to designing clinical trials for the combination of radiotherapy and immunotherapy. First, the safety of these potential combinations needs to be established. As these combinations have the potential to result in delayed toxicities, the standard 3+3 and continual reassessment models may not be appropriately suited to these purposes. Some investigators have proposed that the time-to-event continual reassessment method is more suitable to clinical trials with agents that might result in acute and late toxicities^8^. Second, it is not certain which dose and fractionation of radiotherapy or which site(s) of disease should be radiated to elicit the optimal immune response. Third, the dosing of immune checkpoint inhibitors, and the sequence to use them with radiotherapy has not been established. Regardless, a trial of ipilimumab with radiotherapy for melanoma and prostate cancer is currently accruing (NCT01449279, NCT01689974, NCT01557114, NCT01565837, NCT 01497808, NCT00861614), and others testing PD-1/PD-L1 inhibitors with radiotherapy are in development. We eagerly await the results of these explorations of the potential therapeutic synergy with radiotherapy and immune checkpoint blockade with the goal of improving patient outcomes in locally advanced and metastatic cancers.

**Figure 1:**
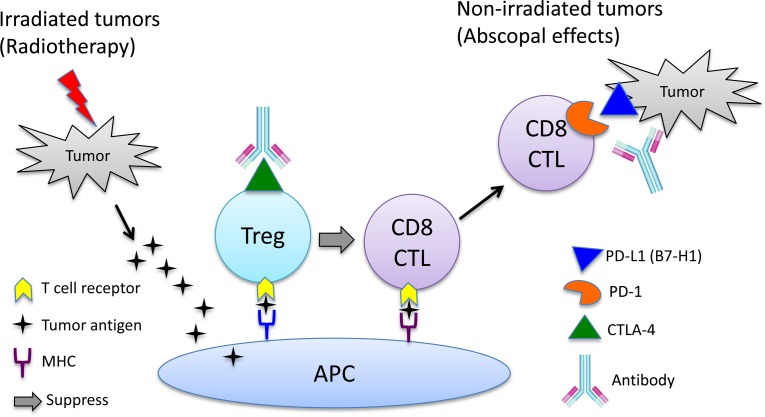
Potential mechanism of action of combined radiotherapy and immunotherapy Antigen presenting cells (APC) take up tumor antigens released from tumor cells destroyed by radiotherapy to activate CD8 T cells. The antitumor function of activated CD8 T cells (CTL) are suppressed by regulatory T cells (Treg), checkpoint molecules expressed by T cells (CTLA-4 and PD-1) and by tumor cells (PD-L1 /B7-H1). Immunotherapy with antibodies blocking the immune suppression function of Treg cells and checkpoint molecules restores the antitumor activity of CTLs capable of rejecting tumor cells that are not in the radiation field ( an abscopal effect).
